# Impact of sit-stand desks at work on energy expenditure and sedentary time: protocol for a feasibility study

**DOI:** 10.1186/s40814-016-0071-1

**Published:** 2016-07-18

**Authors:** Eleni Mantzari, Katrien Wijndaele, Soren Brage, Simon J. Griffin, Theresa M. Marteau

**Affiliations:** 1 Behaviour and Health Research Unit, University of Cambridge, Cambridge, UK; 2 MRC Epidemiology Unit, University of Cambridge, Cambridge, UK

**Keywords:** Sit-stand desks, Standing desks, Height-adjustable desks, Sitting, Standing, Energy expenditure, Sedentary behaviour

## Abstract

**Background:**

Prolonged sitting, an independent risk factor for disease development and premature mortality, is increasing in prevalence in high- and middle-income countries, with no signs of abating. Adults in such countries spend the largest proportion of their day in sedentary behaviour, most of which is accumulated at work. One promising method for reducing workplace sitting is the use of sit-stand desks. However, key uncertainties remain about this intervention, related to the quality of existing studies and a lack of focus on key outcomes, including energy expenditure. We are planning a randomised controlled trial to assess the impact of sit-stand desks at work on energy expenditure and sitting time in the short and longer term. To reduce the uncertainties related to the design of this trial, we propose a preliminary study to assess the feasibility and acceptability of the recruitment, allocation, measurement, retention and intervention procedures.

**Methods:**

Five hundred office-based employees from two companies in Cambridge, UK, will complete a survey to assess their interest in participating in a trial on the use of sit-stand desks at work. The workspaces of 100 of those interested in participating will be assessed for sit-stand desk installation suitability, and 20 participants will be randomised to either the use of sit-stand desks at work for 3 months or a waiting list control group. Energy expenditure and sitting time, measured via Actiheart and activPAL monitors, respectively, as well as cardio-metabolic and anthropometric outcomes and other outcomes relating to health and work performance, will be assessed in 10 randomly selected participants. All participants will also be interviewed about their experience of using the desks and participating in the study.

**Discussion:**

The findings are expected to inform the design of a trial assessing the impact of sit-stand desks at work on short and longer term workplace sitting, taking into account their impact on energy expenditure and the extent to which their use has compensation effects outside the workplace. The findings from such a trial are expected to inform discussions regarding the potential of sit-stand desks at work to alleviate the harm to cardio-metabolic health arising from prolonged sitting.

**Trial registration:**

ISRCTN44827407

## Background

Recent years have witnessed an increased awareness of the detrimental effects of sedentary behaviour (i.e. any waking behaviour characterised by low energy expenditure (≤1.5 METs) and low muscle contraction levels, while sitting or reclining [1–3]). Prolonged sitting time has been shown to increase the risk of obesity, weight gain, diabetes, some cancers, cardiovascular disease and premature mortality, as well as mental health problems [4–9]. These effects are generally shown to be independent of the amount of time spent in moderate-to-vigorous physical activity [1, 10–18]. This implies that one could meet or exceed the recommended daily physical activity levels but still be at risk if the rest of the day is spent sitting. Sedentary behaviour accumulated in prolonged uninterrupted bouts (e.g. bouts >30 min) is considered especially problematic, being associated with less healthy cardio-metabolic profiles compared to interrupted sitting, independent of total sitting time [19–22]. The time spent in sedentary behaviour is increasing rapidly on a global scale [23–26] and is expected to continue to do so without intervention [23], rendering it a critical target for public health action [27].

Working-age adults in high-income countries spend approximately 8–9 h a day in sedentary behaviour [28, 29], which is mainly accumulated in three domains: the workplace, at home during leisure, and transport [30]. The majority of all sitting time, however, is gathered at work [31]. Indeed, jobs are becoming increasingly less active and more sedentary [32], with working adults spending often more than two thirds of their time at work sitting [31, 33, 34]. Not only are office workers sedentary most of their time at work, they also tend to sit for prolonged, uninterrupted periods of time. Of further concern is that those who are sedentary for a large proportion of their working day do not compensate by increasing their physical activity levels and/or reducing their sedentary behaviour during leisure time [31, 35, 36]. Reducing and/or breaking up sitting time at work could substantially attenuate the risk of metabolic and cardiovascular disease among office workers [19, 20]. Given that office workers are one of the largest occupational groups [37, 38], decreasing their sedentary behaviour could have impactful population health benefits, making them an important target for preventative approaches [2, 29]. In line with this, recently published expert guidelines advise those working in desk-based occupations to increase standing time and light activity at work by 2–4 h a day [39], although the quality of the evidence that has led to these recommendations is generally low and caution has been advised to policymakers about issuing guidance regarding sitting time reductions as a stand-alone public health intervention [40].

In the past, most existing work-based interventions had focused on increasing levels of physical activity [29, 30]. In recent years, however, there has been a marked interest in identifying ways to reduce occupational sedentary time and promote breaks in sitting. One promising intervention is to alter the workplace environment by providing adjustable sit-stand desks, which allow individuals to work in sitting or standing positions [41]. The occupational ergonomics literature has focused on sit-stand desks for decades [42–44] and has shown that their use can decrease musculoskeletal discomfort without affecting (and sometimes even increasing) work productivity [43, 45–50]. Recently, the use of such desks for reducing prolonged workplace sitting has received new interest, with a number of studies attempting to assess their effectiveness [27, 45, 51–61]. Most of these suggest that installation of sit-stand desks can lead to reduced workplace sitting time and increased standing [27, 51, 52, 54–57, 62–64] (for reviews, see Torbeys et al. (2014) [65] and Neuhaus et al. (2014) [66]), although a couple have failed to find similar effects [53, 60]. One study also reported improvements to high-density lipoprotein (HDL) cholesterol [51] with the use of such desks. However, as concluded by recent reviews [41, 67, 68], the quality of the relevant evidence is low due to most studies’ reliance on small sample sizes and use of low-quality research designs, including the lack of randomisation.

A small number of relevant randomised trials have been conducted [45, 54, 57–59, 61, 62, 69], which, although provide valuable information regarding the use of sit-stand desks work, have some critical limitations leaving key uncertainties regarding the intervention’s impact on sedentary behaviour. These arise from unreliable measurement of sedentary behaviour, weak study designs and lack of consideration of wider behavioural effects of sit-stand desks. For example, some existing trials have used measures of sedentary time that are subjective [45, 62], inadequately validated [54] or unable to distinguish activity in different postures (i.e. sitting vs standing) [54, 58]. Furthermore, results from some trials do not allow observed effects to be attributed to sit-stand desks due to the use of multicomponent interventions [59, 61, 64] or presence of possible confounders. For example, in one trial participants were informed of and reminded weekly of the intervention’s goal to replace 50 % of workplace sitting with standing. Having this knowledge and receiving these weekly prompts could have influenced sitting time thus confounding the impact of sit-stand desks [54]. In addition, reliable estimates of the potential for sit-stand desks to have compensation effects, i.e. greater non-working sitting time and lower energy expenditure and/or increased energy consumption from food and drinks, are precluded by methodological limitations in the few studies that have examined this. These include the use of subjective [57] or invalidated measures to assess sedentary behaviour outside the workplace [54].

Importantly, none of the existing trials have assessed key outcomes relevant to the use of sit-stand desks at work. The first of these includes energy expenditure. Recently, sit-stand desks have been marketed as a means to burn more calories, with alleged benefits to weight loss. However, as pointed out in the aforementioned expert guidelines [39] and confirmed by a recent systematic review of the impact of sit-stand on health-related outcomes, including energy expenditure [70], the existing evidence for such claims is equivocal. The few studies that have assessed energy expenditure in adults with the use of sit-stand desks (for review, see Tudor-Locke et al. (2013) [71]) have been conducted in the laboratory, with small sample sizes, over short periods of time and not part of interventions to reduce sitting. The use of sit-stand desks in the workplace could potentially encourage increased movement, as well as standing during non-working hours. They could also, however, lead to decreased activity during non-work hours (i.e. compensatory behaviour) due to possible fatigue arising from increased standing at work. Such effects cannot be captured in short-duration laboratory-based studies. Quantifying precisely the potential size of the impact of using sit-stand desks at work on overall energy expenditure—in addition to workplace sitting time changes which might have acute physiological effects independent of energy balance—is essential to gauge whether the intervention is likely to have cardio-metabolic health impacts in the short and longer term. It would also allow assessment of ‘compensatory behaviour’.

The second key outcome not assessed by most existing trials is the impact of sit-stand desks on longer term sedentary behaviour. Inclusion of longer term assessments is essential for estimating the sustainability of any observed benefits [66]. Only one trial, currently being conducted, has planned long-term assessments [69]. However, the proposed sample size of this trial is very small (30 participants; 10 participants in each group, which might be further reduced by the potential attrition rates at the longest follow-up assessment). Hence, the quality of the evidence generated by this study is expected to be limited.

In addition, most existing trials (apart from the study by Dunstan et al. (2013) [59], which assessed a multicomponent intervention) have not measured outcomes related to health and cardio-metabolic profiles, which are necessary for the precise evaluation of the potential health benefits of using sit-stand desks at work [29]. Indeed, it was recently concluded that there are substantial evidence gaps to allow for an understanding of the potential of such desks to enhance cardiovascular health benefits by reducing sedentary time [70].

Finally, it is currently unknown whether the potential effects of sit-stand desks on sitting time depend on users’ socio-economic status and therefore the potential for such desks to reduce or increase health inequalities. Although relevant studies are scarce, there is some evidence to suggest that sedentary behaviour is socially patterned. For example, higher levels of TV-viewing have been associated with lower socio-economic status [72, 73] while higher workplace sitting time is associated with higher socio-economic status [73, 74]. We note, however, that existing studies have not focused on the impact of socio-economic status on sitting time specifically among office-based employees. There is a need to determine whether sit-stand desks have the potential to reduce any differences in sitting time arising as a function of socio-economic position among office-based employees and in turn whether they can help reduce the health inequalities that potentially follow from these differences.

We are planning a randomised controlled trial to address the aforementioned uncertainties and extend current knowledge regarding the effectiveness of sit-stand desks at work in reducing overall sedentary behaviour. The planned trial will assess the impact of sit-stand desks at work on energy expenditure and sitting time in the short and longer term, while incorporating a more robust research design than existing studies, including a large sample size and a more valid and reliable measure of the time spent in different body postures. It will also include an assessment of key cardio-metabolic outcomes and will assess whether any effects of sit-stand desks on sitting time are modified by socio-demographic variables, including users’ socio-economic status.

Prior to conducting the aforementioned trial, there is a need to reduce key uncertainties related to its design. These include the following: (i) the feasibility of recruiting eligible participants; (ii) the feasibility of delivering the intervention to participants, i.e. of installing sit-stand desks in their workspaces; (iii) the practical issues associated with delivering the intervention, including the suitability of workspaces for desk installation (e.g. dimensions of available space and room/furniture configuration) and the potential need for storage space for existing and acquired desks; (iv) the number of each desk type^1^ needed for the trial, which depends on potential participants’ preferences for desk mounts vs full desks, as well as the match between their preferences and workspace suitability; and (v) participants’ acceptability of sit-stand desks. To reduce these uncertainties, we are initially proposing a preliminary study with the aim of assessing the feasibility of the procedures for recruitment, measurement, and intervention delivery of the aforementioned randomised controlled trial.

### Aim and objectives

The aim of the current study is to assess the feasibility of conducting a randomised controlled trial of the impact of sit-stand desks at work on energy expenditure and sedentary time. The specific objectives of the study are to:

1. Assess the feasibility of recruiting eligible participants into the trial, by estimating and describing:
  (i) The proportion of eligible participants who are interested in taking part in the trial
  (ii) The baseline characteristics of eligible participants who are interested in participating in the trial, to judge the likelihood of recruiting a sample varied in the potential effect modifiers of interest (BMI, SES, age, gender)
  (iii) The expected recruitment rate
  (iv) The number of recruitment sites (organisations) needed to achieve the target sample size for the main trial
2. Estimate the number of desk mounts and full desks needed for the main trial, by describing:
  (i) The proportion of eligible participants preferring desk mounts vs full desks
  (ii) The proportion of eligible participants with workspaces that permit installation of their desk preference
3. Explore people’s preference regarding the location for the baseline and follow-up assessments (home vs workplace vs clinical research facility)
4. Assess the feasibility and practicalities associated with delivering the intervention (i.e. installing the sit-stand desks)
5. Explore the circumstances under which desks are used in standing mode and the factors that affect desk use
6. Estimate retention and loss to follow-up rates
7. Explore the acceptability of the intervention
8. Explore the acceptability of the outcome assessments and study procedures
9. Assess the variability of outcomes, to inform sample size calculations for the planned trial

## Methods

### Setting and context

The proposed feasibility study will be conducted within two organisations. The first is the Cambridge University Hospitals, National Health Service (NHS) Foundation Trust^2^, which includes two hospitals in Cambridge, UK: Addenbrooke’s Hospital and The Rosie Hospital.

The NHS is one of the largest employers in the world and is the biggest in Europe, with over 1.6 million staff [75]. The cost to the NHS of staff absence due to poor health has been estimated to be £2.4 billion a year. In September 2015, the Chief Executive of NHS England announced a major drive to improve the health and wellbeing of health service staff, which includes initiatives aimed at establishing and promoting physical activity [76].

The second organisation is a private genomics company that specialises in the development, manufacturing and marketing of integrated systems for the analysis of genetic variation and biological function. The company’s headquarters are in the USA but have recently branched out into Cambridge, UK, where approximately 500 employees work across three sites. The study will be conducted in one of the three sites where most office-based employees are based.

## Design

**Fig. 1:**
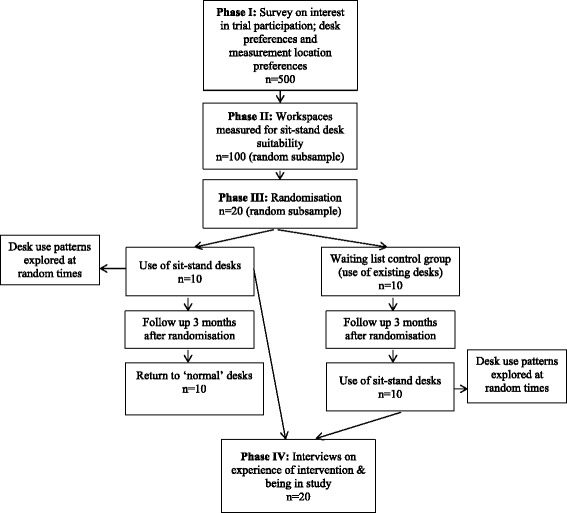
Study overview and participant flow

The proposed feasibility study will consist of four phases (phases I–IV) (Fig. 1). Each phase will address different aims and employ a variety of methods for doing so, including:

- A survey (phase I)
- Workspace auditing (phase II)
- A cross-over randomised component (phase III)
- Direct observations (phase III)
- Qualitative interviews (phase IV)

### Participants

Participants will be office-based employees of two companies in Cambridge, UK.

#### Inclusion criteria

- Work at least 0.6 full-time
- Spend at least 70 % of a working week performing desk-related activities

#### Exclusion criteria

- Are already using sit-stand desks
- Do not have use of their own personal desk
- Have musculoskeletal disorders that make prolonged standing inadvisable
- Have chronic illnesses that prevent prolonged periods of standing
- Are planning to be absent from the workplace for more than 14 working days during the study period
- Are pregnant

For phase I, 500 eligible employees will be targeted. Phase II will involve 100 participants. Both phases III and IV will involve 20 participants.

Eligible participants will be identified and recruited through (i) employment databases and invited via letter/email and (ii) adverts in local newsletters and flyers posted within the buildings of target organisations.

### Sample size

No formal sample size calculations are produced for this feasibility study. Sample sizes for each phase (see Fig. 1) are chosen based on resources. However, it is possible to determine the precision with which certain parameters can be estimated with the sample sizes chosen for each of the study phases.

To achieve the target of 500 individuals completing phase I, the survey will be circulated to approximately 1400 individuals. Although in organisational research the average response rate from individual respondents is 53 %, we have based our estimate on the conservative expected rate of 35 %, which is the average survey response rate for organisational respondents [77–79].

Based on the average interest rates reported in previous studies assessing the impact of sit-stand desks on sitting time, the expected proportion of participants interested in taking part in a trial of sit-stand desks at work is 37 %. With 500 participants, the 95 % confidence intervals around an estimate of 37 % would be from 33 to 41 %.

Based on the average recruitment rates reported in previous studies assessing the impact of sit-stand desks on sitting time, the expected recruitment rate is 33 %. With 500 participants, the 95 % confidence intervals around an estimate of 33 % would be from 29 to 37 %.

The average attrition rate between baseline and follow-up reported in previous studies assessing the impact of sit-stand desks is 10 %. With 20 participants, the 95 % confidence intervals around an estimate of 10 % would be from 2 to 33 %. The maximum attrition rate reported in previous relevant studies is 14 %. With 20 participants, the 95 % confidence intervals around an estimate of 14 % would be from 3.5 to 38 %.

### Procedure

#### Phase I

During phase I, approximately 1400 eligible participants from across organisations will be invited to complete an online survey, of whom we aim 500 to complete the survey, to assess the following: (a) their interest in participating in a trial aiming to assess the use of sit-stand desks at work; (b) their preferences for different desk types (full desks vs desk mounts); and (c) their preference regarding the location (home vs workplace vs clinical research facility) for the baseline and follow-up assessments.

The survey will include a brief description of the trial and will ask people to indicate their willingness to participate, via a yes/no response. The proposed duration of the future full trial, the design of which this feasibility study aims to clarify, is 6 months, Due to resource restrictions, the duration of the randomised component of the proposed feasibility study will be 3 months. To gauge any potential differences in interest to participate as a function of the trial duration, all participants will be asked to respond to two relevant questions: one enquiring about their interest in a trial lasting 3 months and one enquiring about their interest in a trial lasting 6 months. The order in which these questions appear will be randomised. Participants will also be asked to indicate their preference regarding potential locations for the assessments to take place (home, workplace, or clinical research facility).

The survey will also include a brief description, with pictures, of the two different types of sit-stand desks available: full desks and desk mounts that are installed on top of existing desks. Links to videos demonstrating how each desk type works will also be provided.

The survey will also include questions relevant to participants’ demographic information, including age, gender, level of education, BMI, smoking status, salary band, and position within the organisation.

#### Phase II

Of the participants indicating an interest in taking part in the trial during phase I, 100 chosen at random will be visited by a researcher who will assess their workspaces for desk installation suitability. To do this, the researcher will measure the following: (a) the dimensions of the workspace, (b) the dimensions of existing desks, and (c) the dimensions of any available (empty) space. The researcher will also record (through photographs and notes) the configuration of the space (i.e. shape of the room) and of the furniture within that space, making note of any obstacles that might inhibit desk installation and/or use (e.g. shelves that would obstruct sit-stand desks being put into standing mode).

These measurements and records will be compared against measurements involving the space and room configurations needed to successfully install each desk type, identified from measuring acquired samples of each desk type.

#### Phase III

Twenty randomly selected participants of those whose workspaces will be measured during phase II will be randomised to either the use of sit-stand desks (full desks or desk mounts depending on their preference and workspace limitations) at work for 3 months or a waiting list control group. Those allocated to the waiting list control group will continue using their existing desks and will be offered sit-stand desks 3 months later.

#### Intervention

The intervention will comprise the provision of sit-stand desks at work for 3 months. Participants will be offered one of the two desk types according to their preferences and workspace restrictions: desk mounts or full desks. This is in line with the procedures adopted in previous studies [54, 69] and is expected to maximise the probability of desk use. Desk mounts involve a device that is installed on top of a conventional workplace desk often by means of a clamping arm. The device facilitates regular transitions between sitting and standing postures, predominantly while performing computer-based activities. It can be placed in standing mode via an easy upward pulling motion that lifts the display unit(s) and objects placed on the work surfaces. With full sit-stand desks, the entire surface area of the desk (and all items on it) can be adjusted to standing mode.

To enhance desk use, participants will also be given the following: (i) a demonstration of how their desk works, delivered in person by a researcher; (ii) written instructions on how to use the desks; (iii) information on the correct ergonomic posture; (iv) information on the benefits of standing, reducing sitting and breaking up sitting time; (v) guidance on gradually building up standing time; (vi) recommendations relating to the amount of time to increase standing and reduce sitting; and (vii) information on how the desks are used by others.

#### Randomisation

Following baseline assessments, participants will be randomised to the use of sit-stand desks at work or a waiting list control group, who will receive sit-stand desks 3 months later. The randomisation will be determined by a statistician independent from the research team, with the assistance of computer software.

#### Assessments

Of those randomised during phase II, 10 participants, five from the interventions group and five from the waiting list control group, will complete the baseline and follow-up assessments.

Based on their preference of location, participants will be visited by a researcher at their home, or place of work, or invited into a clinical research facility for all baseline assessments to be made prior to randomisation. The measurements will include anthropometric (height, weight, fat mass, fat-free mass, waist and hip circumference) and blood pressure measurements and collection of a blood sample to measure total cholesterol, HDL, triglycerides and HbA1C. Participants will also be asked to complete a questionnaire to assess musculoskeletal discomfort and other health symptoms, ability to work, presenteeism, absenteeism and job satisfaction, quality of life and domain-specific sedentary behaviour.

They will also be fitted with a combined heart rate and movement sensor (Actiheart) on the chest and asked to complete either a stepping test, if assessed at home or work, or a treadmill test if assessed in the clinical research facility. They will also be fitted with a thigh-based accelerometer (activPAL) to measure baseline sitting standing and stepping time and sitting and standing patterns. Both devices will be worn for 24 h/day for seven consecutive days. Participants will also be required to complete a brief daily log with wake and sleep times, work hours and any device removal, as well as their food intake. Participants will receive instructions on devices and log use. Devices and logs will be collected by research staff at the end of the 7-day period.

Follow-up assessments will occur 3 months after baseline. One week prior to this, participants will be visited by a research assistant who will fit them with the inclinometers and monitors, to be worn 24 h/day for seven consecutive days. Data from these will be used to assess the impact of the intervention on sitting and standing time, as well as sitting and standing patterns, and physical activity energy expenditure. Participants will also be asked to complete the daily logs. Seven days later, depending on participants’ location of preference, devices and logs will either be collected by research staff, who will visit participants at home or at work, or will be returned by participants visiting the clinical research facility. At the same time, participants will provide a blood sample and have their anthropometric and blood pressure measurements taken. They will also be asked to complete the same questionnaires previously completed at baseline.

After the follow-up assessments, participants will receive a personalised report with feedback on their health status, levels of physical activity, fitness and sedentary behaviour and information on how these have changed over time. If any of the assessments indicate abnormalities, participants will be notified of their results by the study clinician (SJG) in the first instance and then instructed to visit their general practitioner.

During phase III, all participants given sit-stand desks will be asked to complete short weekly video and/or paper-based diaries to record the circumstances under which they choose to use the desks to stand, as well as the barriers and facilitators to standing. At random times, participants will also be directly observed in their workplaces by a researcher to record their behaviour, including the different tasks undertaken while standing vs sitting. The content of the diaries and the results from the observations will be analysed to create a list of the possible factors that facilitate and inhibit desk use. This information will be used in designing the main trial to maximise the possibility of desk use.

#### Phase IV

Participants offered sit-stand desks will be interviewed about their experiences of using sit-stand desks, in order to explore the circumstances under which they chose to stand both at work and in other contexts; their attitudes towards standing in a predominantly sitting environment, i.e. the workplace; and the acceptability of the intervention and of the study procedures. Employer representatives will also be interviewed, in order to explore the acceptability of sit-stand desks at the organisational level. Interviews will be semi-structured and will last approximately 30 min. An interview schedule will be designed based on the existing literature and in collaboration with an expert qualitative researcher with experience in research within the field of physical activity and public health. The interview schedule will be pre-piloted on a small number of employees within the target organisation who currently use sit-stand desks.

### Outcomes and measures

#### Phase I

The following outcomes will be assessed via an online questionnaire:

- Proportion of eligible participants interested in taking part in a 3-month trial on the use of sit-stand desks at work
- Proportion of eligible participants interested in taking part in a 6-month trial on the use of sit-stand desks at work
- Demographic characteristics
  - ° Age
  - ° Gender
  - ° BMI
  - ° Smoking status
  - ° Salary band
  - ° Highest educational qualification
  - ° Position within company
- Proportion of participants preferring desk mounts vs full desks
- Proportion of participants preferring to be assessed at home vs work vs the clinical research facility

#### Phase II

- Proportion of workplaces suitable for use of full desks and desk mounts assessed via workplace auditing and recording
- Proportion of workspaces permitting participants’ choice of desk to be installed

#### Phase III

- Practicalities of delivering the intervention, assessed by recording:
  - ° The time lapse between ordering the desks from the manufacturer and their delivery
  - ° Any permissions needed to install each desk type
  - ° The feasibility of training research staff to install desks
  - ° The feasibility of one person (a trained research assistant) installing each desk without help
  - ° The amount of time needed to install each desk
  - ° Any problems associated with delivering the desks to participants
  - ° Any problems installing each desk type
  - ° The practicalities associated with removing existing desks (applicable only when using full desks)
- Circumstances and factors affecting desk use assessed via direct observations and diaries/logs
- Proportion of participants dropping out between randomisation and the 3-month follow-up
- Trial-related outcomes, assessed at baseline (before randomisation) and at 3-month follow-up:
  - ° Physical activity energy expenditure estimated via Actiheart^3^ monitors
  - ° Sedentary behaviour measured using activPAL inclinometers^4^:
    - Sitting time during (a) working hours (workplace sitting time) and (b) all waking hours (total sitting time)
    - Sitting patterns (number of sit-to-stand transitions; sitting time accrued in prolonged bouts (≥30 min)) during (a) working hours (workplace sitting patterns) and (b) all waking hours (total sitting patterns)
  - ° Cardio-metabolic related outcomes:
    - BMI calculated from weight and height
    - Weight measured using a scale
    - Height measured using a stadiometer
    - Fat mass and fat-free mass measured via a spectroscopy device
    - Blood pressure, measured via an electronic monitor
    - Waist-hip circumference measured using a tape measure
    - Plasma total cholesterol, HDL, triglycerides and HbA1C, measured via non-fasting blood tests
  - ° Musculoskeletal discomfort measured using the Nordic Musculoskeletal Questionnaire [80]
  - ° Ability to work, work productivity, presenteeism, absenteeism and job satisfaction measured using the Work ability index [81], the Stanford presenteeism scale [82] and Measure of job satisfaction [83]
  - ° Domain-specific sedentary behaviour measured using the SIT-Q-7d, a domain-specific last-7-day sedentary behaviour questionnaire [84]
  - ° Health symptoms, including neck pain, headache, back pain, fatigue, eye strain and loss of concentration, measured using a checklist
  - ° Health-related quality of life measured using the Euro-Quality of Life 5 (EQ-5D-5L) [85]
  - ° Food and drink consumption measured using a daily food log

#### Phase IV

All outcomes assessed via qualitative interviews:

- Experiences of using desks, including factors perceived as affecting desk use, issues with desk use (contextual, practical, emotive or others) and adverse consequences (work, health or otherwise related)
- Experiences of other intervention components
- Company representatives’ perceptions of using sit-stand desks
- Acceptability of intervention
- Acceptability of assessments and burden
- Acceptability of study procedures

#### Other measures

- Proportion of participants dropping out between phases

#### Data analysis

The main analysis of this study will include descriptive statistics of feasibility and acceptability outcomes, including recruitment and attrition rates. We will also calculate differences between the average change from baseline in energy expenditure and sitting time between the intervention and control groups to estimate possible effect sizes from which to power the main trial.

Analysis of the anonymised data gathered through the semi-structured interviews will be conducted following the principles of the Framework method [41].

#### Research governance

The study is funded by a grant from the Department of Health Policy Research Program (Policy Research Unit in Behaviour and Health [PR-UN-0409-10109], the Medical Research Council [Unit Programme number MC_UU_12015/3] and the British Heart Foundation [Intermediate Basic Science Research Fellowship grant FS/12/58/29709 to KW]. The funders have no role in the study design, data collection or analysis; decision to publish; or preparation of the manuscript. Ethical approval was obtained by the University of Cambridge Psychology Department Research Ethics Committee (reference number PRE.2015.100; date of approval: November 18, 2015). Management, data storage and analysis will be conducted at the Behaviour and Health Research Unit, Primary Care Unit, Department of Public Health and Primary Care, University of Cambridge.

## Discussion

The time spent in sedentary behaviour is rapidly increasing in middle- and high-income countries and is expected to continue to do so without intervention [23]. Given the health implications of prolonged and uninterrupted sitting, this creates a huge public health problem. Reducing and/or breaking up sitting time at work could substantially attenuate the risk of disease among office workers [19, 20], who are sedentary a large proportion of their day. Given that office workers are one of the largest occupational groups in high-income countries [37, 38], reducing their sedentary behaviour could have important public health implications, making them an important target for intervention.

Most existing interventions to reduce workplace sitting time have focused on changes to the physical environment of workplaces, policy changes and information and counselling [67]. Although strategies falling under the latter two categories have produced inconsistent effects, there is some evidence to suggest that those involving workplace physical changes and specifically the use of sit-stand desks are effective [67]. However, the quality of this evidence is low [41, 67, 68] and key uncertainties remain regarding the use of sit-stand desks at work, including their impact on energy expenditure (in and outside of work) and sitting time in the longer term.

The current feasibility study is designed to finalise the design and conduct of a future, full-scale trial to assess the impact of sit-stand desks at work on energy expenditure (in and outside of work) and sitting time in the short and longer term, in a robust design using objective, reliable and valid measures of energy expenditure and sitting time. Its primary purpose is to address key design uncertainties for the trial, including the feasibility of recruiting eligible participants, their preferences for full sit-stand desks vs desk mounts, the suitability of workplaces for installing each desk type, the practicalities associated with delivering and installing the desks, and to explore the options regarding the location for conducting the assessments (home vs work vs clinical research facility). The qualitative component of the study will allow for exploration of any issues surrounding the acceptability of sit-stand desks from the perspective of the users, as well as of the employers, and of the factors (contextual and personal) that influence desk use. It will also allow for exploration of the acceptability of the study procedures and assessment methods.

The full trial, the design of which the proposed feasibility study aims to clarify, is expected to extend current knowledge regarding the effectiveness of sit-stand desks at work in reducing sedentary behaviour. By focusing on questions regarding the desks’ impact on energy expenditure at work and outside, as well as short and longer term sitting time, using a rigorous design, including mixed methods and robust measures, the trial will provide essential information regarding the intervention’s likely cardio-metabolic health impacts in the short and longer term, which are currently lacking [70]. The findings from this trial are, therefore, expected to inform discussions regarding the potential of sit-stand desks at work to improve outcomes related to the development of risk factors for diseases arising from prolonged sitting.

## Footnotes

1 In the planned trial, participants will be given the option between a desk mount (a device that is installed on top of existing desks) and a full desk, depending on their needs and workspace restrictions.

2 NHS Foundation Trusts are semi-autonomous organisational units within the National Health Service in England. They have a degree of independence from the Department of Health. As of March 2014, there were 147 NHS Foundation Trusts [86].

3 The Actiheart is a compact, chest-worn device that simultaneously records heart rate (HR) and activity (ACC). By combining both activity and heart rate, the accuracy of the energy expenditure calculation is substantially improved. Evidence suggests a good level of agreement between free-living energy expenditure estimates derived through the Actiheart and the doubly labelled water (DLW) method (ICC = 0.81 with no systematic bias) [87–89].

4 The activPAL inclinometer is secured to participants’ thigh and continuously records acceleration, including the gravitational component, allowing for precise inference of the beginning and ending of each bout of sitting or lying (horizontal thigh), standing and stepping at a variety of speeds. The device has been shown to be valid, reliable and sensitive to change [90–94]. It can be waterproofed and therefore worn 24 h/day, resulting in less misclassification between sitting and non-wear time. It is currently the only device that can be used to acquire postural information reliably in free-living situation [95, 96].
